# SUST Bangla Emotional Speech Corpus (SUBESCO): An audio-only emotional speech corpus for Bangla

**DOI:** 10.1371/journal.pone.0250173

**Published:** 2021-04-30

**Authors:** Sadia Sultana, M. Shahidur Rahman, M. Reza Selim, M. Zafar Iqbal

**Affiliations:** Department of Computer Science and Engineering, Shahjalal University of Science and Technology, Sylhet, Bangladesh; National Institutes of Health, UNITED STATES

## Abstract

SUBESCO is an audio-only emotional speech corpus for Bangla language. The total duration of the corpus is in excess of 7 hours containing 7000 utterances, and it is the largest emotional speech corpus available for this language. Twenty native speakers participated in the gender-balanced set, each recording of 10 sentences simulating seven targeted emotions. Fifty university students participated in the evaluation of this corpus. Each audio clip of this corpus, except those of Disgust emotion, was validated four times by male and female raters. Raw hit rates and unbiased rates were calculated producing scores above chance level of responses. Overall recognition rate was reported to be above 70% for human perception tests. Kappa statistics and intra-class correlation coefficient scores indicated high-level of inter-rater reliability and consistency of this corpus evaluation. SUBESCO is an Open Access database, licensed under Creative Common Attribution 4.0 International, and can be downloaded free of charge from the web link: https://doi.org/10.5281/zenodo.4526477.

## Introduction

While communicating, people try to understand each other’s content of speech as well as the active emotion of the speaker. This is depicted by their body language and speech delivery. The study of emotion recognition is important to perceive human behaviors and their relationships both for social studies and human-computer interaction (HCI). It is also important to understand some physiological changes in humans. Research on Speech Emotion Recognition (SER) has been drawing increasing attention of researchers since the last two decades. The first requirement of a functional SER system is to develop a corpus containing useful emotional contents. Studies show that emotional expressions vary from culture to culture [1, 2]. For example, in the Japanese culture, expressing some strong emotions, like anger, are considered to be anti-social [3], whereas this may be different for other cultures. A specific language does not only represent some alphabetic symbols and rules, it also points to a specific culture. For cross-language experiments of emotions, the researchers need different emotional speech corpora for different languages. Researchers have attempted to classify emotions based on a model trained on one language and tested on other languages but the performance was always mediocre in all cases. Few examples are English vs French [4]; English vs German [5]; Chinese vs German and Italian languages [6], and so on. The results show that the SER models performed best for the particular languages on which those were trained on. The motivation of creating SUBESCO is to develop a separate dataset for the Bangla language to facilitate the construction of SER as using a non-Bangla dataset to train Bangla SER may yield a poorer recognition rate.

In recent years, a lot of researches have been conducted on SER for other languages [7–9]. For Bangla language, there is still the lacking of a useful linguistics resource for emotion recognition although there are a few natural speech corpora available for this language [10–12]. But, natural speech corpus is not suitable for speech emotion recognition because all the sounds are recorded in the neutral mode. To study different types of emotions, a corpus has to consist of a high number of, ideally equally distributed, speech utterances for each emotion. An emotional audio corpus is also important for speech synthesis to produce speech with emotional contents [13]. Development of such an emotional corpus is considered to be relatively expensive as professional speakers are needed to express natural-like emotional speeches. Also, a high-quality audio lab is required for recordings to preserve the clear spectral details in the speeches [14] for scientific analysis. The ultimate goal of this study was to build a validated emotional speech corpus as a linguistic resource that will be useful for prosodic analysis of emotional features and emotion recognition for Bangla Language. Researchers can use this dataset to train ML models for classifying basic emotions from Bangla speech. This also will allow carrying out exciting linguistic analyses in comparing Bangla to other related regional languages, e.g., Hindi, Punjabi.

## Related works

Though a few attempts [15] have been made for developing resources for speech emotion analysis for Bangla, they are limited to a few hundreds of utterances. This scope limits the development of an SER based on deep learning approaches, which often needs thousands of training inputs. Recently a group of researchers have developed a small dataset of 160 sentences to implement their proposed method of Speech Emotion Recognition [16]. The acted emotions were happy, sad, angry, and neutral; this dataset with 20 speakers lacks perceptual evaluation data. The most important feature of SUBESCO is that this is the only human validated, gender-balanced emotional speech corpus for Bangla, to date. Literature survey shows a number of mentionable relevant researches done on the development and evaluation of emotional corpus for other languages. American English has a rich collection of versatile linguistic resources in the field of emotional analysis researches. RAVDESS [17] is an audio-visual multi-modal emotional corpus built for American English which has recently been released. It consists of 7356 recordings delivered by 24 professional actors and validated by more than 250 validators. The simulated emotions for this corpus are calm, happy, sad, angry, fearful, surprise, and disgust. Another remarkable emotional corpus IEMOCAP [18] was developed for this language a decade before RAVDESS was built. IEMOCAP contains speech, facial expressions, head motion, and hand gestures for happiness, anger, sadness, frustration and neutral state consisting of approximately 12 hours of data which was validated by 6 listeners. EMOVO [13] speech dataset was built for the Italian language where 6 actors simulated the six emotions of disgust, fear, anger, joy, surprise and sadness. Two different groups of 24 listeners validated the corpus for the human perception test. A database of German emotional speech [14] developed by Burkhardt et al. consists of 800 sentences recorded from the voices of 10 German actors. There are seven emotions neutral, anger, fear, joy, sadness, disgust, and boredom acted out by the speakers. 20 subjects participated in this evaluation task. James et al. developed an open-source speech corpus [19] for 5 primary emotions and 5 secondary emotions consisting of total 2400 sentences for the New Zealand English. There were 120 participants for the subject evaluation of the corpus. An Arabic speech corpus [20] of 1600 sentences was developed by Meftah et al. associating five emotions neutral, sadness, happiness, surprised, and questioning acted out by 20 speakers. Later, it was evaluated [21] by nine listeners to perform a human perception test. There is an emotional speech corpus [22] of 12000 Indian Telegu emotional speech utterances involving eight emotions anger, compassion, disgust, fear, happy, neutral, sarcastic, and surprise. It used 25 listeners to experiment human perception ability of the recorded audios. Recently similar researches have been done for Chinese Mandarin [23], Bahasa Indonesia [24], Greek [25], Danish [26], and several other languages. Depending on the purpose of emotional corpus some researches involve multiple languages [27]. There are some emotional databases that were built using recorded data from human conversations [28, 29], while some are also developed using synthesis systems [30]. Sometimes an emotional corpus involves crowd sourcing for recording evaluation [31]. Ververidis and Kotropoulos [32] have reviewed some important emotional speech databases for different languages.

## Ethics declaration

This corpus has been developed as part of a Ph.D. research project in the Department of Computer Science and Engineering of Shahjalal University of Science and Technology, Sylhet, Bangladesh. Permission was granted by the Ethical Committee of the University to involve human subjects in the research work for the development of the corpus. Written consent was also obtained from all the speakers to publish their voice data and photographs. There were also paid human volunteers involved in data preparation and evaluation experiments. They also gave their written consent to publish their evaluation results and information, if necessary.

## Scope of the database

The developed corpus consists of voice data from 20 professional speakers where 10 of them are males and 10 are females (age: 21 to 35, mean = 28.05 and SD = 4.85). Audio recording was done in two phases with 5 males and 5 females participating in each phase. The gender balance of this corpus has been ensured by keeping an equal number of male and female speakers and raters. There are seven emotional states recorded for 10 sentences. Five trials were preserved for each emotional expression. Hence, the total number of utterances involves 10 sentences × 5 repetitions × 7 emotions × 20 speakers = 7000 recordings. Each sentence length has been kept fixed at 4 sec removing only the silences preserving the full words. The total duration of the database is: 7000 recordings × 4 sec = 28000 sec = 466 min 40 sec = 7 hours 40 min 40 sec. Considering the number of audio clips and the total length of the corpus, at present this is the largest available emotional speech database for Bangla language. A summary of the database is given in Table 1.

**Table 1:** Database summary.

Year of production	2019
Used Language	Standard Bangla
Corpus type	Acted
File type	Audio only
File format of audio clips	.wav
Sampling rate	48KHz
Number of Speakers	10 males and 10 females
Age groups of speakers	20-30 and 30-40
Number of emotions	Seven
Emotion states:	Anger, Disgust, Fear, Happiness, Neutral, Sadness, Surprise
Number of sentences	10
Total number of audio clips	7000
Unit level of audio clips	Sentence
Number of words	50
Number of vowels in the text	7
Number of consonants in the text	23
Number of other phonemes in the text	6 Diphthongs and 1 nasalization
Average duration per clip	4s
Software	Audacity
Total duration of the corpus	28000 sec = 7 hours 40 min 40 sec
Number of validators in each phase	50
Subject evaluation recognition rate	71-80%

## Selection of emotion categories

The most challenging task of emotion recognition is to find the distinguishing features of target emotions. Researchers are still searching for a consensual definition of emotion. Since only voice data is being considered here for the data base design, for correct emotion recognition it is necessary to consider the emotional states which have intense impacts on voice data. For the corpus development, the set of six fundamental emotions [33]: anger, disgust, fear, happiness, sadness, surprise were considered along with neutral emotional state. These are probably the most frequently occurring emotions in all cultures around the world [34]. Neutral can be compared to the Peaceful or Calm state. For this dataset, all the speakers had to simulate these seven emotions for the target 10 sentences. This corpus consists of an equal number of recorded audio clips for all emotions, indicating that the corpus has balanced data in terms of desired emotional states.

## Type of emotion: Acted or real?

Databases of emotional speech corpus may be classified into three types [35] based on the nature of the speech collected: speeches collected in-the-wild, simulated or acted emotional speech database, and elicited emotional speech database. In type 1, real-life emotional speeches are collected from a free and uncontrolled environment for analysis. For example, speeches collected from customer services, call centers, etc. But, the problem is that in-the-wild datasets have no ‘ground truth’, that is, there is no way to know the intended emotion of the speaker at the time of capture which is important for ML models. This kind of dataset also involves some copyright and legal issues. For this reason, they are often unavailable for public use. Type 2 database is developed by collecting acted or simulated speeches. Trained actors are asked to deliver speeches in different emotional states for predefined texts. Most of the available emotional speech databases are acted or simulated. The problem of these types of databases is that sometimes emotions are exaggerated and fail to represent naturally experienced emotions authentically [36]. For controlled scientific experiments, balanced data recorded in a laboratory environment are needed [37] which can be achieved by type 2 database. Type 3 is an elicited emotional speech database in which emotions are induced in speakers using some context. This is not acted in the sense that speakers are provoked to show the real emotions rather than training them to express acted emotions. These kinds of speeches can be collected from some talk shows or reality shows. There are some challenges to build such a database. For example, few emotions like fear and sadness are ethically not permitted to be induced in speakers. Moreover, in some cases, speakers may deliberately hide their emotions (e.g. disgust or anger) in public places to avoid social issues. For type 1, speeches collected in-the-wild have no ground truth and the intensities of induced emotions in type 3 databases are too weak. For this research, clear, strong, lab-based expressions were needed so the speech was collected through acting to develop a type 2 database.

## Database preparation

**Fig 1:**
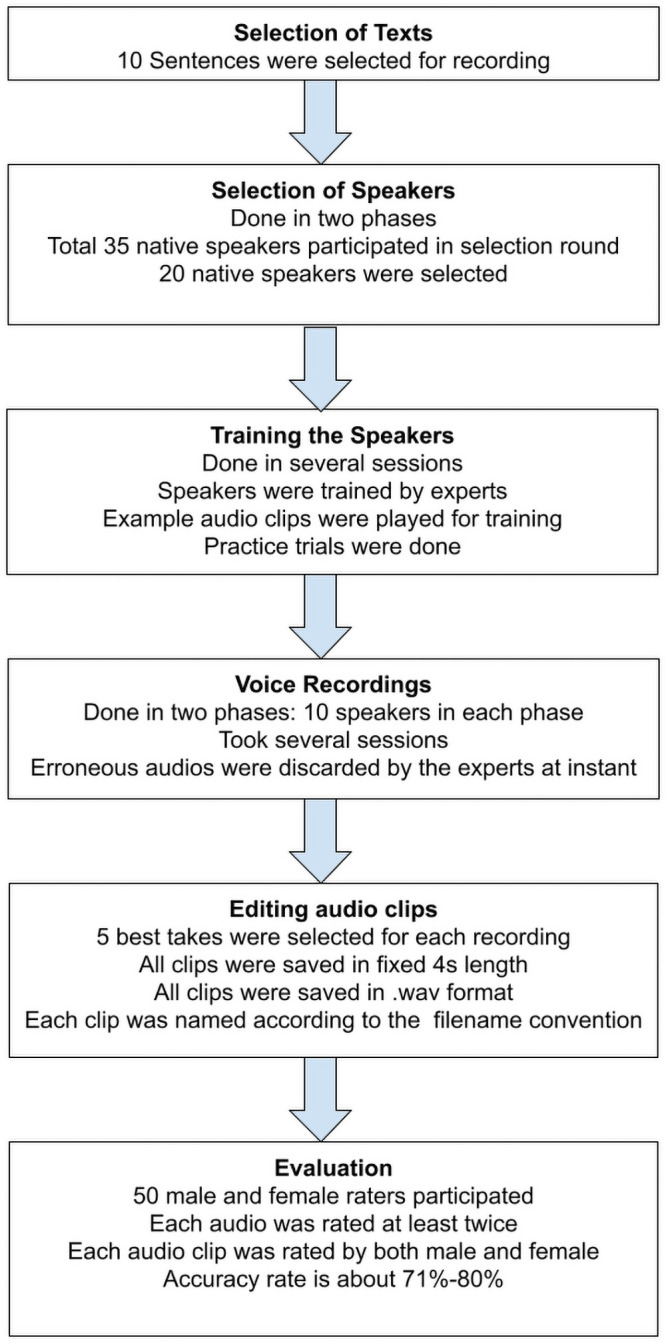
Flowchart of preparation of SUBESCO.

There were several steps involved in the development of SUBESCO. Fig 1 shows all the steps involved in the preparation of the database.

### Context of the corpus

Standard Bangla language has been considered for preparing the text data for developing the emotional corpus. Initially, a list of twenty grammatically correct sentences were made which can be expressed in all target emotions. Then, three linguistic experts selected 10 sentences amongst them for database preparation. One expert is a scholarly Professor of Department of Computer Science and Engineering and has been involved in different Bangla NLP related projects funded by the University and the Govt. of Bangladesh. Other two are Professors of Bangla Department from two different universities. After a trial session it was confirmed that all the aforementioned sentences could be pronounced neutrally. The sentences are structured and kept as simple as possible so that actors can easily memorize and express them. This text data is phonetically very rich which is also important for any language-related research. The selected text dataset consists of 7 vowels, 23 consonants and 3 fricatives covering all major 5 groups of consonant articulation, 6 diphthongs, 1 nasalization (ঁ) of Bangla IPA. The texts of utterances are represented here with two types of transcription, phonemic and phonetic. In phonemic transcription the texts have been represented using the words tier of B-To-BI (Bengali Tones and Break Indices System) [38] transcription and for phonetic transcription the texts have been transcribed with the help of the list of Bangla IPA [39]. In both cases, the phonological model InTraSAL [40], designed by linguistic Professor Sameer Ud Dawla Khan, were followed. Phonemic and Phonetic transcription along with English translations of all the sentences are given below:

Sentence 1:

Bangla: েমৗমািছর চাক েদেখ কুকুরিট েঘউেঘউ করেছ

English: The dog is barking, seeing the beehive

Phonemic: mowmachir cak dekhe kukurTi ghew ghew korche

Phonetic: mowmat̲ɕ^h^iɹ t̲ɕak d̪ek^h^e kukuɹti ɡ^h^ew ɡ^h^ew koɹt̲ɕ^h^e

Sentence 2:

Bangla: তুিম সব উলটপালট কের িদেয়ছ

English: You have messed it all up

Phonemic: tumi SOb uloTpaloT kOre diecho

Phonetic: t̪umi ʃɔb ulotpalot kɔɹe d̪iet̲ɕ^h^o

Sentence 3:

Bangla: েস েকানিকছু না বেলই চেল েগেছ

English: He/she left without saying anything

Phonemic: Se konokichu na bOley cOle geche

Phonetic: ʃe konokit̲ɕ^h^ hu na bɔlej t̲ɕɔle get̲ɕ^h^e

Sentence 4:

Bangla: েতামােক এখিন আমার সােথ েযেত হেব

English: You have to come with me right now

Phonemic: tomake Ekhoni amar Sathe zete hObe

Phonetic: t̪omake εk^h^oni amaɹ ʃat̪^h^e d̲ʑt̪e hɹbe

Sentence 5:

Bangla: েতামার কাজটা করা িঠক হয়িন

English: You shouldn’t have done it

Phonemic: tomar kad̲Ta kOra Thik hOYni

Phonetic: t̪omaɹ kad̲ʑta kɔɹa t^h^ik hɔḙni

Sentence 6:

Bangla: ইঁদুর ছানাটা হািরেয় েগল

English: The mouse pup is lost

Phonemic: iMdur chanaTa harie gelo

Phonetic: i~d̪ur t̲ɕ^h^anata haɹie ɡelo

Sentence 7:

Bangla: এখন পৰ্শ্ন করা যােব না

English: Questions cannot be asked now

Phonemic: Ekhon prOSno kOra jabe na

Phonetic: ɛk^h^on prɔʃno kɔɹa d̲ʑabe na

Sentence 8:

Bangla: দরজার বাইের কুকুরিট দাঁিড়েয় আেছ

English: The dog is waiting outside the door

Phonemic: dOrjar bayre kukurTi daRie ache

Phonetic: d̪ɔrd̲ʑaɹ bajɹe kukuɹti daɾie at̲ɕ^h^e

Sentence 9:

Bangla: একিদন পেরই তার িবেয়

English: She is getting married the day after

Phonemic: Ekdin porey tar bie

Phonetic: Ekd̪in poɹej t̪aɹ bie

Sentence 10:

Bangla: ডাকােতরা ঢাল তেলায়ার িনেয় এল

English: The robbers came with shields and swords

Phonemic: Dakatera Dhal tolOWar nie elo

Phonetic: dakat̪eɹa d^h^al t̪olɔo̭aɹ nie elo

### Selection of speakers

Speaker selection is a very important task in an emotional speech database preparation. Research [36] has shown that real-life emotion recognition rate is higher for non-professional speakers because sometimes professional speakers produce exaggerated emotional expressions while recording. But, for a natural-like noiseless speech database, the speech expressions of professional actors were recorded in a technical sound studio. Professional artists are very comfortable delivering a speech in front of a microphone for recordings, it was easy to instruct them how to accomplish the task. For audition and voice recordings of all speakers, there were three experts, including the first author. Two paid and trained research assistants helped from the process of audition of the speakers to final preparation of the audio clips. The speaker selection and recordings were done in two phases. In the first phase, 10 professional actors (5 males, 5 females) were selected from 20 participants. A few months later in the second phase of voice recording, another group of 10 professional speakers (5 males, 5 females) were selected from 15 participants. There were 3500 recordings done in each phase. All of the participants were native Bangla speakers and involved in different platforms of theatre act. After the training session, in the audition round in front of the experts they were asked to express Sentence 8 simulating all the target emotions. The experts selected the final participants based on the recognisability and naturalness of their speeches. It took a couple of days to select all the participants. After selection, the speakers filled up the personal information forms and signed the consent papers to deliver their speech, in return for a fixed amount of reimbursement. A recording plan was scheduled according to their availability.

### Technical information

The sound recording studio is furnished with acoustic walls and doors that make it anechoic to maintain professional sound studio quality. It is partitioned into a recording room and a control room. The glass partition between the two rooms significantly attenuates the amount of sound passing through. A condenser microphone (Wharfedale Pro DM5.0s) for recording with a suitable microphone stand (Hercules) was provided to the speaker. The average distance between the microphone and the mouths of the speakers was 6 cm. The microphone was connected to a USB Audio Interface (M-AUDIO M-TRACK 2×2, 2-IN/2-OUT 24/192). Two Logitech H390 USB Headphones were also connected to the audio interface so that experts could listen to the speeches at the time of the recordings. Intel(R) Core(TM) i7-7700 CPU @ 3.60Hz 3.60GHz processor was used with an 8.00 GB RAM as the hardware tools and Operating System installed in it was 64-bit Windows 10. There was an HP N223v 21.5 Inch TN w/LED backlight Full-HD monitor display to visualize the audio during recording. The output was recorded as 32-bit mono wav format at a sampling rate of 48 kHz. For recording and editing, an open-source sophisticated software tool Audacity (version 2.3.2) was used.

### Procedure and design

Audio recordings took place in an anechoic sound recording studio which was set up in the Department of Computer Science and Engineering of Shahjalal University of Science and Technology. In the recording room, the speaker was provided a seat and a dialog script which contained all the sentences numbered in sequential order. The same sentence numbering was used in file naming to recognize each recording separately. A condenser microphone was set up in front of the seat and the microphone level could be adjusted so that the speaker could easily deliver his or her speech while sitting on the seat. Professional artists are familiar with the famous Stanislavski method [14] to self-induce the desired emotions. No extra effort was needed to induce the target emotions in them. They were requested to portray the emotions to make the recordings sound like natural speeches as much as possible. Speakers were allowed to take as much time as needed to prepare themselves to express the intended emotions properly. Two trained research assistants were sitting in an adjacent control room and they could visually observe the recorded speech on a computer display. They could also hear the recordings via headphones connected to a controlling device which was also connected with the microphone of the recording room. They could give feedback to the speakers, with predefined gestures, through the transparent glass partition between the two rooms. Some erroneous audio clips were discarded instantaneously and others were saved on the computer for post-processing, if agreed by the experts. The recording was done for one speaker at a time. Each speaker took several sessions to deliver his or her speech to simulate all sentences for the target emotion categories.

### Post-processing

Post-processing of the recordings was done to make all data suitable for prosodic analysis and automatic feature extraction. Every speaker needed 9-12 hours for recording and gave 700-1000 takes. From those takes, the best five tracks were selected and the number of the final tracks for every person was 350. After recordings, backup copies of all voice clips were saved as uncompressed wav format files in an external SATA hard disk to avoid unexpected loss of data. During editing, the silence was removed from the audio tracks and they were cut into 4s audio clips without trimming off any word. Peak normalization of -1dB was applied to all the recordings to preserve the natural loudness and distortion-free playback. They were renamed so that each file could be identified separately. After selection, editing, and renaming, all the recordings were reviewed again. They were then stored separately in 20 folders which were named according to the speakers’ names.

### Filename convention

All the speakers were assigned serial numbers. The sentences were also documented in a predefined sequential order. Serial numbers of speakers were given according to the recording schedules assigned to them. For example, speaker number M_01 indicates to the male speaker whose voice was recorded first of all, M_02 means the person is the second male participant in the schedule, and so on. After editing each audio file was given a separate and unique file name. The filename is a combination of numbers and texts. There are eight parts in the file name where all the parts are connected by underscores. The order of all the parts is organized as: Gender-Speaker’s serial number-Speaker’s name-Unit of recording-Unit number- Emotion name- Repeating number (last two parts) and the File format. For example, the filename F_12_TITHI_S_10_SURPRISE_TAKE_5.wav refers to: female speaker (F), speaker number(12), speaker’s name (TITHI), sentence-level recording(S), sentence number (10), emotional state (SURPRISE), take number (TAKE_5) and the file extension (.wav). The coding is represented in Table 2 for a better understanding.

**Table 2:** Filename convention.

Gender	F(Female), M(Male)
Speaker Number	01 = 1^st^ Speaker,…,10 = 10^th^ Speaker
Speaker Name	First Name Written As Text
Sentences	S_1 = েমৗমািছর চাক দেখ কুকুরিট েঘউ ঘউ কর ছ
	S_2 = তুিম সব উলটপালট কের িদেয়ছ
	S_3 = েস েকানিকছু না বেলই চেল েগেছ
	S_4 = েতামােক এখিন আমার সােথ েযেত হেব
	S_5 = েতামার কাজটা করা িঠক হয়িন
	S_6 = ইঁদুর ছানাটা হািরেয় েগল
	S_7 = এখন পৰ্শ্ন করা যােব না
	S_8 = দরজার বাইের কুকুরিট দাঁিড়েয় আেছ
	S_9 = একিদন পেরই তার িবেয়
	S_10 = ডাকােতরা ঢাল তেলায়ার িনেয় এল
State of Emotion	NEUTRAL, HAPPINESS, SADNESS, ANGER, FEAR, DISGUST, SURPRISE
Repetition	TAKE_1 = 1^st^ Take, ….., TAKE_5 = 5^th^ Take

## Corpus evaluation

**Fig 2:**
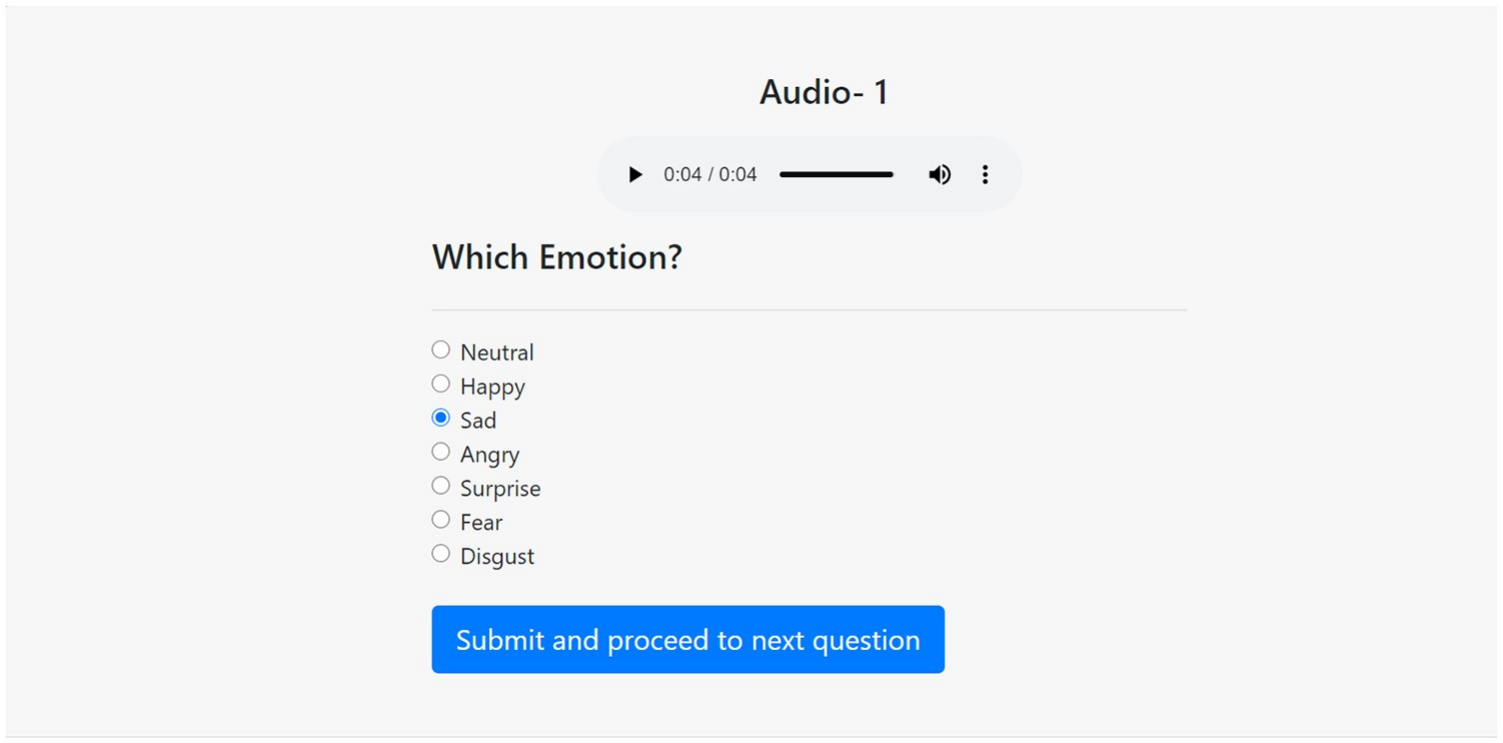
Forced-choice rating task screen used in perceptual rating for Phase 1.

The main purpose of the corpus evaluation is to find out to what extent an untrained listener can correctly recognize the emotion of the recorded audios. A higher recognition rate indicates a higher quality of recordings. The whole task of the evaluation was done in two phases. *Phase* refers to a test-retest done in different periods of time to accomplish the evaluation of the corpus. Human subjects were involved to accomplish the task. There were 25 males and 25 females, with a total of 50 raters for each phase. Each rater evaluated a set of recordings in Phase 1, then after a week’s break, rated the same set of recordings in Phase 2. In the first phase, the raters evaluated all seven emotions. In the second phase, the emotion Disgust was not considered while the other six emotions were considered, mainly because it was to investigate the fact that Disgust was causing a confusion with other similar emotions. The intention was not to exclude Disgust from the dataset, rather the aim was to release the complete dataset for public use. In this case, Phase 2 can be considered as a separate study related to Disgust which can facilitate the users to decide whether they should include it or not for their audio-based research as studies have found that to be more likely a visually-expressed emotion [41]. The same set of 50 evaluators participated in both phases because the goal was to find out the effect of some important factors on the recognition rate by comparing the results from both phases. All of the raters were Shahjalal University students from different disciplines and schools. All of the participants were both physically and mentally healthy and aged over 18 years at the time of the evaluation. None of them participated in the recording sessions. All of them were native Bangla speakers and they could efficiently read, write and understand the language. The raters were not trained on the recordings to avoid any bias in their perception ability. A management software was developed including a user interface for the overall management of the corpus evaluation task. Listeners could access the interface through Wi-Fi and wired LAN connections in the same building. For the first phase of the evaluation, 25 audio sets were prepared where each set contained pseudo-randomly selected 280 stimuli. For the second phase, those audio sets contained 240 audio files as Disgust was removed from them. Each audio set was assigned to two persons, a male rater and a female rater to ensure that each audio clip is rated twice and by both genders in each phase. Each of the raters were provided with a seat and a computer with a display in front of him or her. Prior instructions were given to them on how to accomplish the task. Before starting the evaluation process, an expert explained to all the participants the whole task, providing some sample audios from an existing emotional speech database of other languages. After starting the experiment, all the selected audios were presented on a screen one after another, in front of each rater. On the screen, each audio was displayed with a track number and all the emotion categories were listed under it. There was a submit button at the bottom of the screen. A rater could select only one emotion, for a single audio, which he thought to be the best match. An example display of the evaluation process is shown in Fig 2 for Phase 1. Similar screen was used for Phase 2 excluding the option for Disgust. If the speaker’s intended emotion had matches with the rater’s selected emotion it was considered as the correct answer, ‘incorrect’ otherwise. A correct answer had a score of 1 and an incorrect answer had a score of 0. During the evaluation, each time the rater submitted an opinion it was automatically converted to 1 or 0. The rater’s selected emotion was also preserved for later analysis. After completing the evaluation, the proportion of the total number of correctly recognized emotions to the total number of audios reviewed by the rater was calculated as ‘percent correct score’ which is the ‘raw hit rate’. The ‘percent correct score’ was calculated for each emotion per rater. As is well known, only the proportion statistics alone is not sufficient to represent the overall consistency and reliability of any implementation and analysis [42], and it cannot be used directly for performance analysis. Rather, it has been used to calculate several statistical methods to prove the reliability and consistency of the implemented corpus and its evaluation. Raw hit rate does not correct for false alarms that is why unbiased hit rate was calculated based on Wagner’s formula [43]. The formula corrects the possibility of selecting the correct emotion category by chance or bias due to answer habit [44].

## Reliability of evaluation

The whole task of the evaluation was accomplished in a separate controlled environment classroom so that the students could participate in a noise-free environment and concentrate on the audios. User login and activation deactivation sessions were created in the system for the security of the dataset and to avoid unwanted data from uninvited users. The whole system was managed by the administrator (first author) and a sub-administrator (a research assistant). Before starting, the students were instructed on how to accomplish the task and asked to register with their personal details. It is to be noted that listeners were untrained about the recordings, to avoid any influence of past experience. After registration, each participant’s details were verified and his or her id activated for a certain period of time. They were able to review the selected audios intended for them after completing registration and user login. The audio tracks were shuffled before being played to the raters. The submit button was kept deactivated until the user played the full audio so that the user could not proceed to the next audio without listening to the current audio. After submitting one could not move to the previous recordings so as to eliminate the chance to compare the speeches of the same speaker. Listeners were allowed to save and exit the experiment anytime in case of any discomfort and he or she could restart from the saved session if it still remained activated by the administrator. Raters were given a break of 15 minutes after each 45 minute session to avoid any psychological stress due to the experiment. Once a listener submitted all the questions, he or she was deactivated by the administrator to make the retaking of the experiment not possible. The second phase of evaluation was done after a one week break to avoid any influence by the user’s memory. All the raters were given at least 95% of the previous audio tracks so that the performances of the two phases could be compared point to point. Clips were reshuffled before playing to confirm that those were in a order different from the first phase.

## Results

### Phase 1 evaluation

There were 7000 audio clips in this phase. Each audio was rated twice so that the total number of ratings was 14000 in this phase. The overall raw hit rate for this step is 71% with mean SD = 8.24. Table 3 represents the raw hit rate and unbiased hit rate for each emotion. It can be seen that the highest recognition rate was achieved by Neutral (raw = 85%, SD = 7.6), whereas Happiness achieved the second-highest rate (raw = 77.4%, SD = 9.8). Disgust has the lowest recognition rate (raw = 59.1%, SD = 9.6). From Table 4 it is obvious that the largest confusion occurred between Anger and Disgust (18.4%), which is more than 5% of the total ratings for this phase. More than one fourth Anger emotion audios were incorrectly recognized as Disgust. The emotion Disgust is also largely confused with other emotions like Surprise(11.5%), Neutral (7.6%), and Happiness(5.7%). After taking the feedback of the participants it was clear that they were struggling to discriminate between Disgust and other emotions. Studies show that recognizing Disgust as an emotion, based only on audios is not an easy task [41]. In real-life experience, it may be noticed that Disgust executes some facial expressions like frowning, smirking which gives strong cues in understanding it. Neutral was wrongly classified as Sadness and Disgust for more than 5% of the audios. Another major confusion occurred between Fear and Sadness. Fear was wrongly classified as Sadness in more than 19% of the cases. The majority of wrongly classified emotions for Sadness were recognized as Neutral and Fear. Happiness is confused with Neutral, Sadness, and Disgust with a lesser degree. Still, the results are noticeable. There was also a large confusion between Happiness and Surprise. The reason for the confusion between similar emotions is well explained in a study carried out by Posner et al. [45]. It states that a person’s emotion recognition ability is associated with the person’s prior experience and some internal and external cues. Another study [46] shows that overlapping cognitive schema decreases a person’s ability to discriminate between similarly valencing emotions (e.g. Happiness and Surprise). Rater and speaker gender-based mean raw accuracies are shown in Table 5.

**Table 3:** Raw hit rates (H) and unbiased hit rates (Hu) for emotion categories: Phase 1.

Emotion	H		Hu	
	Mr%	SD%	Mr%	SD%
**Neutral**	85.1	7.6	61.0	10.0
**Happiness**	77.4	9.8	64.2	9.4
**Sadness**	75.8	8.0	52.2	9.1
**Anger**	66.7	8.0	55.2	7.5
**Surprise**	67.9	7.4	50.9	10.1
**Fear**	65.4	7.3	50.9	7.2
**Disgust**	59.1	9.6	31.0	9.8

Mr = Mean hit rate; SD = Standard Deviation

**Table 4:** Confusion matrix for Phase 1 evaluation result.

Emotion	Neutral	Happiness	Sadness	Anger	Surprise	Fear	Disgust	Total
**Neutral**	1702	5	147	8	12	6	120	2000
**Happiness**	127	1548	103	4	81	22	115	2000
**Sadness**	190	32	1516	3	14	188	57	2000
**Anger**	65	6	5	1334	26	46	518	2000
**Surprise**	91	163	17	39	1357	92	241	2000
**Fear**	65	7	387	14	135	1308	84	2000
**Disgust**	183	113	47	218	218	39	1182	2000
**Total**	2423	1874	2222	1620	1843	1701	2317	14000

Rows represent rater-chosen emotions, columns represent speaker-acted emotions.

**Table 5:** Average recognition rates for males and females in Phase 1.

Emotion Name	M SP vs All RT	F SP vs All RT	All SP vs M RT	All SP vs F RT	M SP vs M RT	M SP vs F RT	F SP vs M RT	F SP vs F RT	Mean
**Neutral**	82.6%	87.6%	83.9%	86.3%	81.6%	83.6%	86.2%	89.0%	85.1%
**Happiness**	79.3%	75.5%	75.8%	79.0%	77.2%	81.4%	74.4%	76.6%	77.4%
**Sadness**	71.1%	80.5%	75.3%	76.3%	71.2%	71.0%	79.4%	81.6%	75.8%
**Anger**	72.8%	60.6%	67.8%	65.6%	74.2%	71.4%	61.4%	59.8%	66.7%
**Surprise**	64.7%	71.0%	64.9%	70.8%	63.4%	66.0%	66.4%	75.6%	67.9%
**Fear**	66.4%	64.4%	64.9%	65.9%	66.2%	66.6%	63.6%	65.2%	65.4%
**Disgust**	64.5%	53.7%	55.1%	63.1%	60.6%	68.4%	49.6%	57.8%	59.1%
**Mean**	71.6%	70.5%	69.7%	72.4%	70.6%	72.6%	68.7%	72.2%	71.1%

M = Male; F = Female; SP = Speakers; RT = Raters.

**Fig 3:**
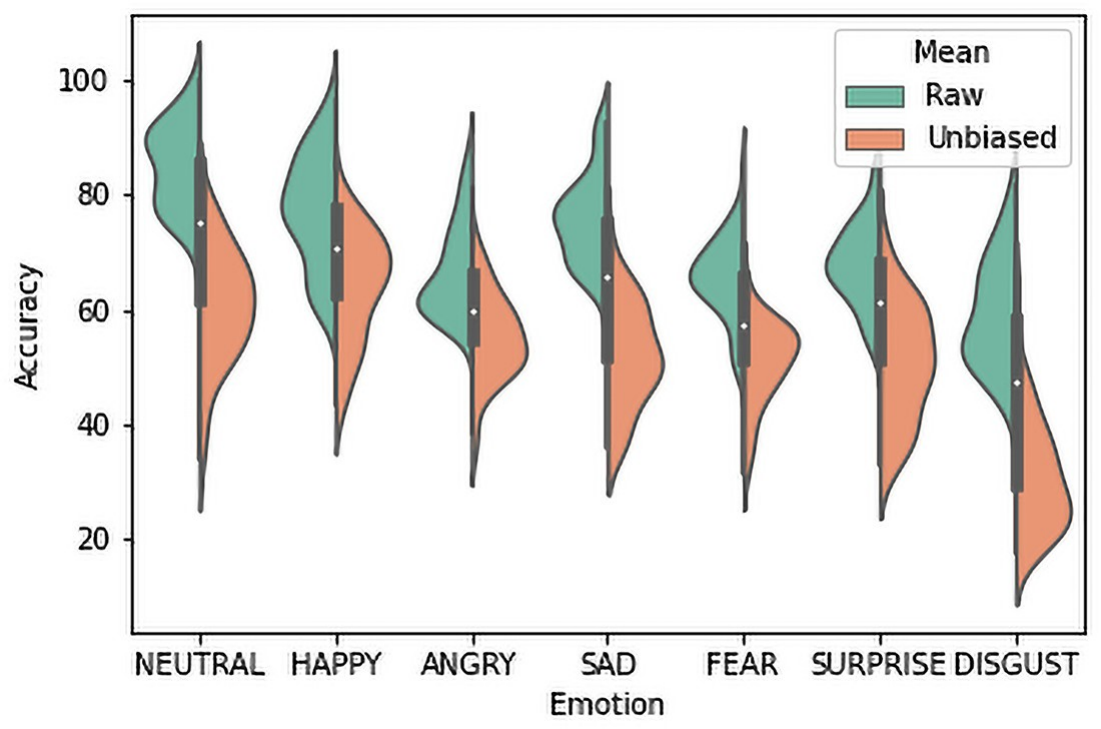
Comparing the raw and unbiased hit rates for Phase 1.

The overall unbiased hit rate for this phase is 52.2% with SD = 9.0. Unbiased hit rates are lower than raw hit rates for all emotion categories since these are corrected scores and they ignore bias; comparison is shown in Fig 3. The rank order for recognition rates is different than that for raw hit rate. Still, Fear and Disgust are the least recognized emotions. The change in rank order is due to the major confusions between the emotions: Happiness and Neutral, Anger and Disgust, Fear and Sadness etc. There were 7 choices for each trial, thus the chance level of perception rate is 15%. Both the raw and unbiased hit scores were above the chance level for all emotion categories. Table 6 shows that all the sentences have achieved fair recognition rates for all target emotions. Neutral has achieved the highest scores almost in all cases for emotion perception evaluation.

**Table 6:** Sentence-wise recognition rates for Phase 1.

Sentence	Neutral	Happiness	Anger	Sadness	Fear	Surprise	Disgust
**Sentence 1**	94.5%	71.0%	44.0%	66.5%	78.0%	70.0%	56.0%
**Sentence 2**	70.5%	68.0%	68.5%	81.0%	49.5%	62.5%	68.5%
**Sentence 3**	76.5%	76.5%	66.5%	87.5%	47.5%	71.5%	61.0%
**Sentence 4**	87.0%	80.0%	79.0%	76.0%	61.0%	48.0%	55.0%
**Sentence 5**	77.0%	74.0%	75.5%	77.5%	57.5%	46.0%	68.5%
**Sentence 6**	83.0%	81.5%	58.0%	80.0%	54.5%	88.5%	54.0%
**Sentence 7**	84.5%	88.0%	80.0%	80.5%	78.5%	56.5%	65.0%
**Sentence 8**	93.0%	73.5%	66.0%	60.0%	80.5%	77.5%	66.0%
**Sentence 9**	89.0%	78.5%	68.0%	81.5%	68.0%	74.0%	56.0%
**Sentence 10**	96.0%	83.0%	61.5%	67.5%	79.0%	84.0%	41.0%

### Phase 2 evaluation

**Fig 4:**
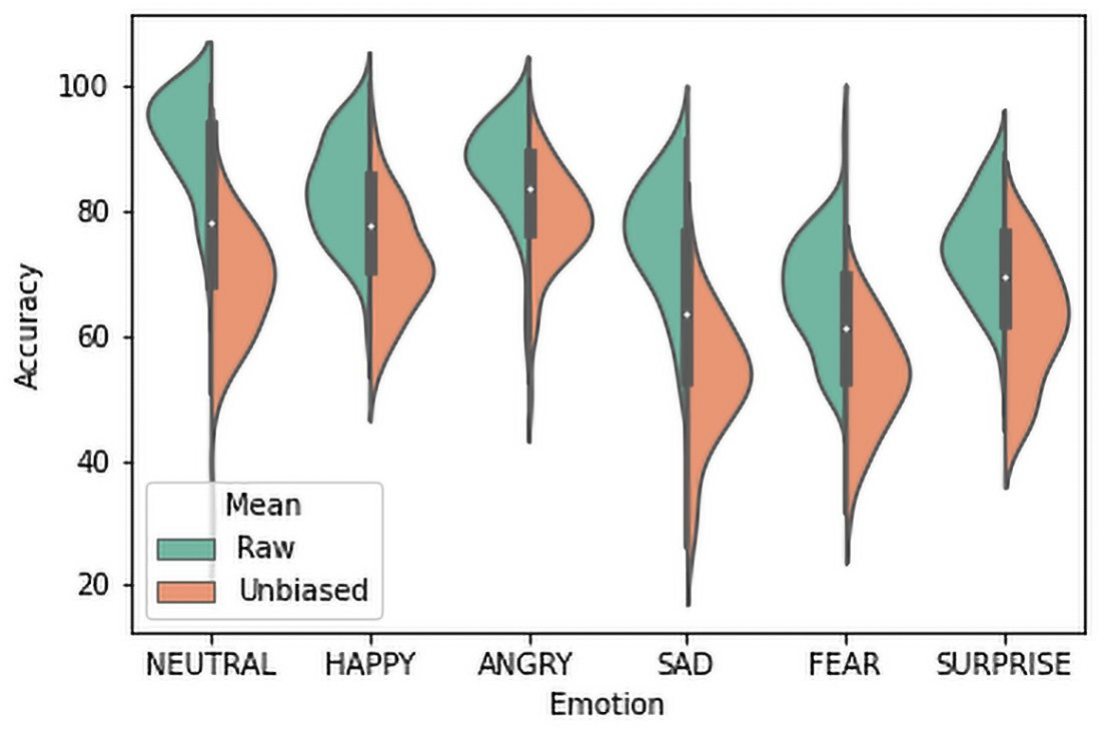
Comparing the raw and unbiased hit rates for Phase 2.

In the second phase, there were 6000 audio clips and the total number of ratings was 12000. After removing Disgust in this phase, interestingly, the mean recognition rate was increased by 80% (SD = 8.4). Table 7 statistics show that there is a sharp rise in the raw hit rate for Anger in this phase, which is more than 20% (66.7% to 87.2%). Recognition rates for Neutral, Happiness, and Surprise also increased by 6.4%, 5.5%, and 6.3% respectively. Other emotions have nearly the same results compared to the previous phase. This confirms the doubt that Disgust was suppressing the overall recognition rate. But, a look at the confusion matrix in Table 8 reveals that still major confusions persisted between Fear and Sadness(14.5%), Neutral and Sadness (8.5%), Fear, and Surprise (6%). In this phase, Neutral has the highest recognition rate (91.5%) whereas Fear has the lowest recognition rate (67.2%) (Table 7). For both phases, female raters have relatively higher recognition scores compared to male raters, for all cases (Tables 5 anmd 9). Gender effects will be discussed more elaborately later in this paper. Rank order for unbiased hit rates is completely different in this phase (unbiased = 68.1, SD = 9.22). Anger has the highest position (unbiased = 78.3, SD = 8.2) due to the removal of large confusion introduced by Disgust. Sadness has the lowest position (unbiased = 53.2, SD = 10.1). Relative positions between Happiness and Neutral, Surprise and Fear are still the same. Since there were six options for each rating to the users the chance level of responses for this phase is 17%. Recognition scores for all emotion categories were higher than 17%. Raw and unbiased hit rates are compared in Fig 4 for Phase 2. Sentence-wise recognition rates for all emotions for this phase is represented in Table 10.

**Table 7:** Raw hit rates (H) and unbiased hit rates (Hu) for emotion categories: Phase 2.

Emotion	H		Hu	
	Mr%	SD%	Mr%	SD%
**Neutral**	91.5	7.9	68.1	10.0
**Happiness**	82.9	8.3	72.1	8.6
**Sadness**	75.4	9.6	53.2	10.1
**Anger**	87.2	7.7	78.3	8.2
**Surprise**	74.2	7.8	62.8	9.7
**Fear**	67.2	8.8	53.5	8.7

Mr = Mean hit rate; SD = Standard Deviation

**Table 8:** Confusion matrix for Phase 2 evaluation result.

Emotion	Neutral	Happiness	Sadness	Anger	Surprise	Fear	Total
**Neutral**	11830	14	110	31	8	7	2000
**Happiness**	129	1657	92	23	84	15	2000
**Sadness**	233	48	1507	8	8	196	2000
**Anger**	128	13	28	1743	44	44	2000
**Surprise**	90	187	31	107	1483	102	2000
**Fear**	87	9	384	40	136	1344	2000
**Total**	2497	1928	2152	1952	1763	1708	12000

Rows represent rater-chosen emotions, columns represent speaker-acted emotions.

**Table 9:** Average recognition rates for males and females in Phase 2.

Emotion Name	M SP vs All RT	F SP vs All RT	All SP vs M RT	All SP vs F RT	M SP vs M RT	M SP vs F RT	F SP vs M RT	F SP vs F RT	Mean
**Neutral**	89.3%	93.7%	90.1%	92.9%	87.8%	90.8%	92.4%	95.0%	91.5%
**Happiness**	85.3%	80.4%	81.7%	84.0%	83.8%	86.8%	79.6%	81.2%	82.9%
**Sadness**	72.1%	78.6%	74.8%	75.9%	71.8%	72.4%	77.8%	79.4%	75.4%
**Anger**	93.3%	81.0%	86.0%	88.3%	93.0%	93.6%	79.0%	83.0%	87.2%
**Surprise**	71.1%	77.2%	72.1%	76.2%	70.2%	72.0%	74.0%	80.4%	74.2%
**Fear**	67.7%	66.7%	66.1%	68.3%	65.2%	70.2%	67.0%	66.4%	67.2%
**Mean**	79.8%	79.6%	78.5%	80.9%	78.6%	81.0%	78.3%	80.9%	79.7%

M = Male; F = Female; SP = Speakers; RT = Raters.

**Table 10:** Sentence-wise recognition rates for Phase 2.

Sentence	Neutral	Happiness	Anger	Sadness	Fear	Surprise
**Sentence 1**	98.5%	77.5%	79.0%	64.0%	82.0%	75.5%
**Sentence 2**	85.5%	77.0%	92.5%	82.5%	50.0%	69.0%
**Sentence 3**	85.5%	77.5%	88%	86.0%	49.0%	78.5%
**Sentence 4**	89.0%	88.0%	93.0%	77.0%	62.0%	60.5%
**Sentence 5**	86.0%	82.0%	92.0%	79.5%	61.5%	56.0%
**Sentence 6**	90.0%	83.0%	74.0%	83.0%	57.5%	91.05%
**Sentence 7**	92.5%	91.0%	90.5%	75.5%	85.0%	65.5%
**Sentence 8**	96.0%	83.0%	90.5%	61.0%	77.0%	81.0%
**Sentence 9**	93.0%	81.0%	90.5%	61.0%	77.0%	81.0%
**Sentence 10**	99.0%	88.5%	83.5%	65.0%	74.5%	86.0%

## Statistical analyses

For the hypothesis tests, the confidence interval was set at 95%, therefore, a null hypothesis was rejected when the probability statistics p-value become less than the critical value =.05. Kappa statistics and Intra-class Correlation (ICC) were carried out to analyze the reliability of the rating exercises. All the test statistics were rounded off to the 2nd decimal and all probability statistics were rounded off to the third decimal. The data was analyzed using the Python programming language. Two-way ANOVA was performed as a further study after evaluation task to investigate the variability and interaction of the main factors which are Gender and Emotion. To perform ANOVA on the data, the raw hit rates for each rater for all emotions categories were considered for both phases. For speaker data, raw hit rate for each emotion category against each speaker’s performance was calculated. Actually, ANOVA was performed on those data after log transformation as normality of distribution was not satisfied. Pairwise comparisons have been executed for post-hoc analysis.

### Inter-rater reliability

Fleiss’ Kappa was used to investigate the inter-rater reliability of the evaluation. It is an adaptation of Cohen’s Kappa which is used when the rater number is more than two. Fleiss’ Kappa requires that each subject should be different and there should be a fixed number of raters for each file and not every rater needs to evaluate all files [47]. In this database, there are 10 sentences, 7 emotions, and 20 speakers for recordings. Thus, the total number of unique utterances is 1400. If the repeating takes are considered as a single file then it can be said that during the evaluation each audio was validated 10 times. It is assumed that N = number of files, n = number of ratings for each file and k = number of categories. Then, for each phase, the calculation of Kappa score involved an ***N*×*n*** matrix, and the total number of reviews was ***N* × *k***. According to the guidelines established by Landis and Kotch [48], Kappa scores < 0 indicate poor agreement, 0.01–0.20 indicate slight agreement, 0.21–0.40 fair agreement, 0.41–0.60 moderate agreement, 0.61–0.80 substantial agreement, and 0.81–1 indicate almost perfect agreement. Phase 1 inter-rater reliability obtained a mean Kappa value of 0.58 which is considered as a moderate agreement between the raters. Mean Kappa value for Phase 2 was 0.69 which indicates a substantial agreement between the raters. It can be inferred that the difference in Kappa scores between the two phases is due to the higher recognition rates in Phase 2. The overall result says that raters’ performances were consistent across the overall evaluation of the audios.

### Intra-class correlation coefficients (ICCs)

The Intra-class correlation coefficient is a reliability index when **n** number of files are rated by **k** number of judges [49]. It reflects both the consistency and measurement agreements between the raters [50]. Several intra-class correlations are used to evaluate inter-rater, intra-rater, and test-retest reliability for different rating trials. As all the raters were not provided with all the audio clips for evaluation, only ICC(1,1) and ICC(1,k) were considered for this case. ICC (1,1) is a one-way random effect, an absolute agreement for a single rater; and ICC(1,k) is a one-way random effect, an absolute agreement for multiple raters. The reported value of ICC(1,1) for this analysis was 0.751 which indicates good reliability of measurements. For ICC(1,k) the obtained value 0.99 indicates excellent reliability for 95% confidence interval according to the guidelines suggested by Koo and Li [51]. It suggests that values < 0.40 indicate poor agreement, 0.40–0.59 fair agreement, 0.60–0.74 good agreement, and 0.75–1 indicate excellent agreement. ICC estimates and their 95% confidence intervals were calculated using open-source statistical package pingouin for Python version 3.6.5. The statistics are presented in the Table 11. Note that the estimator is the same regardless of the presence of interaction effects.

**Table 11:** ICC: One-way random effects model for absolute agreement definition.

	ICC	F	df1	df2	p-value	CI95%
**Single measures**	0.751	151.432	1	98	.000	[0.36, 1.0]
**Average measures**	0.993	151.432	1	98	.000	[0.97, 1.0]

### Normality test

**Fig 5:**
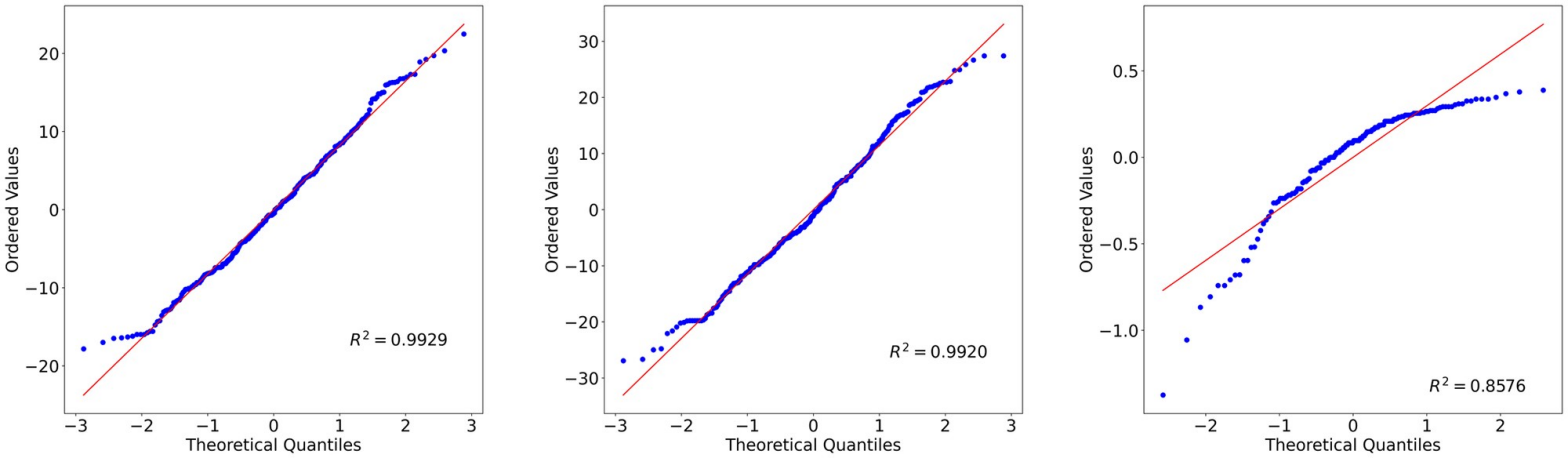
Probability plots for Phase 1 data. (A)Emotion (B)Rater gender (C)Speaker gender data.

**Fig 6:**
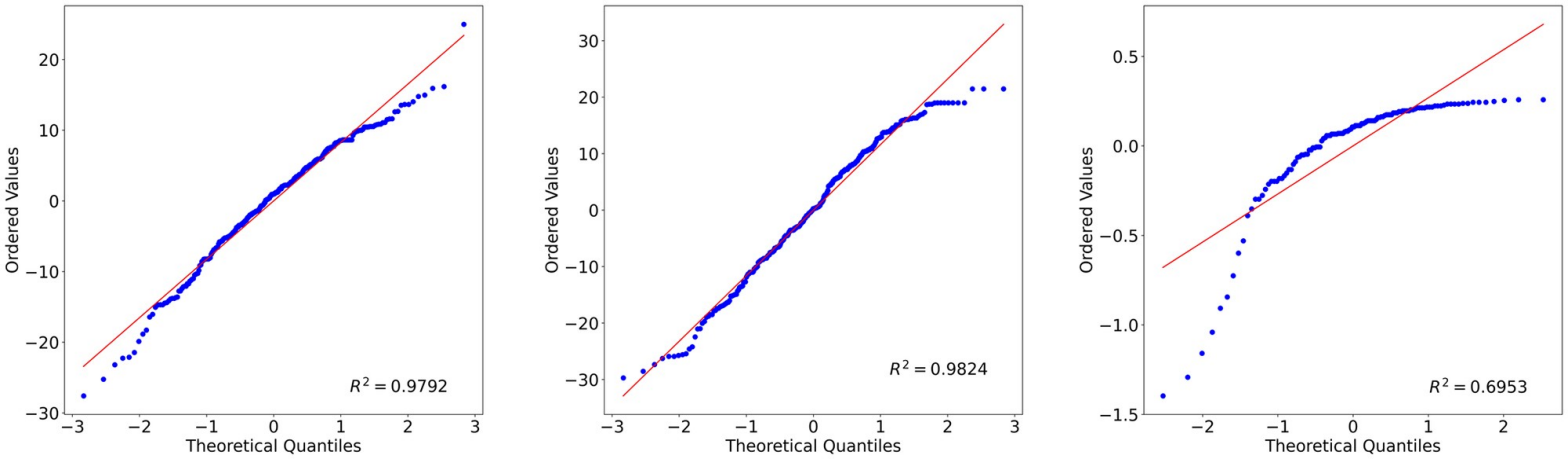
Probability plots for Phase 2 data. (A)Emotion (B)Rater gender (C)Speaker gender data.

Before applying any statistical test on a dataset, it is worth investigating its probability distribution type. The Jarque–Bera and Shapiro-Wilk tests were applied on one-way ANOVA residuals to examine the probability distributions of data based on Rater Gender, Speaker Gender and Emotion. The null hypotheses of these tests assume that the target population is normally distributed. According to the test statistics, they were rejected for both tests except Rater Gender data for Phase 1 for the Jarque-Bera statistics (Table 12). That means the normality assumption was not satisfied for those factors. Figs 5 and 6 present the probability plot distributions of the factors. It can be seen that the distributions are not normal, data points are skewed and removed from the fitted lines. Therefore, log transformation was applied to the data to normalize it. Transformation could not normalize all data except for Phase 1 Rater Gender and Emotion. Still, the Two-way ANOVA was performed as it was assumed that it is robust against normality assumption violation when the sample size is large and homogeneity assumption is satisfied.

**Table 12:** Normality statistics tests for emotion and gender-wise data.

Source	Phase	Jarque-Bera Test					Shapiro-Wilk Test			
		Skew	Kurtosis	JB	prob	Reject H_*o*_	Statistics	df	prob	Reject H_*o*_
**Speaker**	Phase 1	-0.518	3.315	14.65	0.001	Reject	0.94	1	0.000	Reject
**Gender**	Phase 2	-0.518	3.315	14.65	0.001	Reject	0.84	1	0.000	Reject
**Rater**	Phase 1	0.176	2.536	4.95	0.084	Accept	0.99	1	0.018	Reject
**Gender**	Phase 2	-0.286	2.405	8.53	0.014	Reject	0.98	1	0.000	Reject
**Emotion**	Phase 1	-0.518	3.315	14.65	0.001	Reject	0.99	6	0.034	Reject
	Phase 2	-0.518	3.315	14.65	0.001	Reject	0.98	5	0.000	Reject

### Homogeneity test

There are two main assumptions of ANOVA, normality of data and homogeneity of variances. The Levene and Bartlett test were performed on Emotion, Speaker Gender and Rater Gender to test for the homogeneity of their variances. The analysis was carried out for Phase 1 and Phase 2 raw hit rate data. The null hypothesis of the test states that, the population variances are equal across the groups of the variable. For all of the variables the results were not statistically significant and thus confirmed the homogeneity of variances across the groups of the variables. Table 13 represents the details statistics of homogeneity tests.

**Table 13:** Homogeneity test for Gender and Emotion-wise data.

Source	Phase	Levene’s Test				Bartlett’s Test			
		Statistics	df	p-value	Reject H_*o*_	Statistics	df	p-value	Reject H_*o*_
**Speaker**	Phase 1	1.49	1	.225	Accept	0.85	1	.355	Accept
**Gender**	Phase 2	0.12	1	.733	Accept	0.12	1	.732	Accept
**Rater**	Phase 1	1.20	1	.274	Accept	1.25	1	.263	Accept
**Gender**	Phase 2	0.73	1	.393	Accept	0.58	1	.446	Accept
**Emotion**	Phase 1	2.04	6	.060	Accept	9.26	6	.159	Accept
	Phase 2	0.97	5	.436	Accept	3.72	5	.590	Accept

### Two-way ANOVA

Two-way ANOVA was conducted on log transformed data to find out Gender and Emotion interaction effects. It was calculated for the rater and speaker genders separately, for both phases, at the significance level of p < 0.05. The independent variable in all cases was the raw hit rate. For rater gender at Phase 1, the main effect of Gender was statistically significant for emotion perception at the condition of F(1,336) = 11.96, p <.001. Emotion also has a significant main effect on average hit rate where F(6,336) = 58.95, p <.001. Two-way interaction between Gender and Emotion was also significant for F(6,336) = 2.59, p <.05. Table 14 shows the summary results for this phase. For Phase 2, from Table 15 it is seen that the main effect of rater Gender was statistically significant for F(1,288) = 6.58, p <.05. The main effect of Emotion was also statistically significant, where F(5,288) = 53.94, p <.001. But the two-way interaction between Gender and Emotion was not statistically significant, where F(6,336) = 0.11, p >.05. Tables 16 and 17 show the results of a two-way ANOVA between Speaker Gender and Emotion for transformed raw hit rates. The results show that the Speaker Gender does not have statistically significant main effects on perception rates (p >.05). But, Emotion has a statistically significant main effect on the recognition rate for both phases. Where, F(6,126) = 4.27, p <.001 for Phase 1 and F(5,108) = 4.10, p =.001 for Phase 2. There is no statistically significant interaction between Speaker Gender and Emotion. This indicates that, there is no statistically significant evidence that the male speakers performed better for any specific emotion recognition compared to females or vice versa. As the data does not hold normal distribution equivalent non-parametric tests were carried out to see the effect of Gender and Emotion on recognition rate. Similar results were found after conducting those tests, the analysis is discussed in Appendix.

**Table 14:** Descriptive table for two-way ANOVA for Phase 1(for rater gender).

Source	sum_sq	df	mean_sq	F	PR(>F)	eta_sq	omega_sq
**C(Gender)**	0.16	1.0	0.16	11.96	.000	0.017	0.016
**C(Emotion)**	4.59	6.0	0.76	56.95	.000	0.485	0.475
C(Gender)*C(Emotion)	0.21	6.0	0.03	2.59	.018	0.021	0.013
**Residual**	4.51	336.0	0.01	NaN	NaN	NaN	NaN

Dependant variable: Corrected mean hit rate

**Table 15:** Descriptive table for two-way ANOVA for Phase 2(for rater gender).

Source	sum_sq	df	mean_sq	F	PR(>F)	eta_sq	omega_sq
**C(Gender)**	0.08	1.0	0.08	6.58	.010	0.011	0.009
**C(Emotion)**	3.37	5.0	0.67	53.94	.000	0.477	0.467
C(Gender)*C(Emotion)	0.01	5.0	0.00	0.11	.098	0.001	-0.008
**Residual**	3.59	288.0	0.01	NaN	NaN	NaN	NaN

Dependant variable: Corrected mean hit rate

**Table 16:** Descriptive table for two-way ANOVA for Phase 1(for speaker gender).

	sum_sq	df	mean_sq	F	PR(>F)	eta_sq	omega_sq
**C(Gender)**	0.03	1.0	0.03	0.29	.590	0.002	-0.004
**C(Emotion)**	2.25	6.0	0.37	4.27	.000	0.159	0.121
C(Gender)*C(Emotion)	0.76	6.0	0.13	1.44	.204	0.054	0.016
**Residual**	11.05	126.0	0.09	NaN	NaN	NaN	NaN

Dependant variable: Corrected mean hit rate

**Table 17:** Descriptive table for two-way ANOVA for Phase 2(for speaker gender).

	sum_sq	df	mean_sq	F	PR(>F)	eta_sq	omega_sq
**C(Gender)**	0.00	1.0	0.00	0.01	.938	0.000	-0.007
**C(Emotion)**	1.85	5.0	0.37	4.10	.001	0.154	0.115
C(Gender)*C(Emotion)	0.44	5.0	0.09	0.98	.432	0.036	-0.000
**Residual**	9.74	108.0	0.09	NaN	NaN	NaN	NaN

Dependant variable: Corrected mean hit rate

### Post-hoc analysis

ANOVA shows that there is a significant difference between the means of the populations. If the F-score of a factor is statistically significant then further detailed analysis is done using post-hoc analysis. This test is used on the variable that has more than two population means [52]. Moreover, the test assumes that homogeneity of variances assumption is satisfied, and sample sizes are equal for the variables [53]. Pairwise t-test was conducted to compare the means of emotional categories based on raw hit rate for the post-hoc analysis. It is protective against type I error and decreases the chance of type II error. For this data, all pairwise comparisons were done at a significance level of 0.05. Emotion has a significant main effect on emotion recognition. It has more than two samples of population means and it reserves the homogeneity assumption. Post-hoc analysis was applied for pairwise comparisons of different emotions. Test statistics for post-hoc analysis using this method are summarized in Tables 18 and 19 for Phase 1 and Phase 2 respectively. Positive and negative values represent the differences between the means of emotions. For Phase 1, pairwise comparison shows that the mean of Anger is significantly different (p <.001) from the mean of Disgust, Happiness, Neutral, and Sadness. However, Fear and Surprise are not significantly different from Anger. The mean of Disgust significantly differs from that of all other emotions. The mean of Fear did not significantly differ from Anger and Surprise. The mean of Happiness significantly differs from all except Sadness. Neutral also significantly differs from all emotions for the mean. The mean of Sadness significantly differs from all except that of Happiness. Surprise differs significantly from all except Anger and Fear. For Phase 2, the result is quite different from Phase 1. All the comparisons show significant differences except between Sadness and Surprise (p >.05).

**Table 18:** Pairwise comparisons for Phase 1 emotions.

	Anger	Disgust	Fear	Happiness	Neutral	Sadness	Surprise
**Anger**	1	4.268*** [4.0, 11.13]	0.905 [-1.61, 4.25]	−6.005*** [-14.17, -7.07]	−11.02*** [-21.62, -14.95]	−5.360*** [-12.12, -5.5]	-0.816 [-4.16, 1.76]
**Disgust**		1	−3.825*** [-9.53, -2.97]	−11.29*** [-21.42, -14.95]	−14.92*** [-29.33, -22.37]	−10.20*** [-19.61, -13.15]	−6.341*** [-11.55, -5.99]
**Fear**			1	−7.089*** [-15.32, -8.55]	−14.03*** [-22.41, -16.79]	−6.426*** [-13.30, -6.96]	-1.820 [-5.30, -0.26]
**Happiness**				1	−4.530*** [-11.06, -4.26]	0.942 [-2.05, 5.66]	6.108*** [6.32, 12.52]
**Neutral**					1	5.417*** [5.96, 12.98]	12.609*** [14.36, 19.8]
**Sadness**						1	4.514*** [4.22, 11.0]
**Surprise**							1

Statistical test reporting: Mean difference M[95% confidence interval: Lower, Upper]. Asterisks for the p-values are interpreted as:

* is <.05,

** is < 0.01,

*** is <.001.

**Table 19:** Pairwise comparisons for Phase 2 emotions.

	Anger	Fear	Happiness	Neutral	Sadness	Surprise
**Anger**	1	−11.56*** [16.39, 23.28]	2.514* [0.79, 7.09]	−2.675** [-7.37, -1.05]	6.380*** [8.16, 15.67]	7.751*** [9.58, 16.29]
**Fear**		1	−11.267*** [-18.73, -13.06]	−14.52*** [-27.37, -20.72]	−4.740*** [-11.28, -4,56]	−4.241*** [-10.17, -3.63]
**Happiness**			1	−4.817*** [-11.55, -4.75]	4.784*** [4.63, 11.33]	−5.327*** [5.60, 12.39]
**Neutral**				1	8.912*** [12.49, 19.76]	11.41*** [14.12, 20.16]
**Sadness**					1	0.618 [-2.30, 4.34]
**Surprise**						1

Statistical test reporting: Mean difference M[95% confidence interval: Lower, Upper]. Asterisks for the p-values are interpreted as:

* is <.05,

** is < 0.01,

*** is <.001.

## Discussion

The main purpose of this study is to represent SUBESCO, the largest emotional audio dataset for Bangla. It is the only gender-balanced, human validated emotional audio corpus for this language. The overall evaluation was presented using two studies done in Phase 1 and Phase 2. Overall raw hit rate for Phase 1 was 71%, and for Phase 2, it was 80%. If we look at the perceived hit rates for other relevant audio-only datasets including: Arabic database: 80% for 800 sentences [21], EMOVO: 80% for 588 files [13], German database: 85% for 800 sentences [17], MES-P: 86.54% for 5376 stimuli [23], Indonesian speech corpus: 62% for 1357 audios [24], Montreal affective voices: 69% for 90 stimuli [54], Portuguese dataset: 75% for 190 sentences [55], RAVDESS: 62.5% for 1440 audio-only speech [17]; these results confirm that the perceptual hit rate of SUBESCO was comparable to existing emotional speech sets. Unbiased hit rates were also reported along with raw hit rates to address false alarms. A separate study in Phase 2 evaluation was carried out to test the effect of Disgust to facilitate researcher’s selection of Disgust stimuli for their research paradigm. Inter-rater agreement is substantial with 0.58 for Phase 1 and 0.63 for Phase 2, respectively. Also, excellent Intra-class correlation scores were achieved: ICC = 0.75 and 0.99 for single and average measurement respectively. Fleiss’ Kappa and ICC confirms the reliability and consistency of the built corpus. It can be said that SUBESCO was successfully created and evaluated with a fair enough recognition rate which makes it a very useful resource for further study on emotional audio for Bangla. The set of stimuli can be used by the researchers of other languages for prosodic analysis. It can also be used in cognitive psychology experiments related to emotion expression.

Detailed analysis are presented of the emotional categories based on recognition rates. Also the effects of gender on the perception of emotion were observed. The outcomes of the experiments and statistical analyses suggest the following outcomes. Neutral has the highest recognition rate and it is easy to recognize. Happiness and Sadness have relatively higher recognition rates than others except for Neutral. There has been a difficulty in recognising Disgust based on audio only. However, adding a facial expression (video) might help recognizing it better. Disgust is likely to be confused with Anger, as the recognition rate of Anger improved noticeably after removing it in the second phase. Surprise also achieves a better score without Disgust. Fear has a low recognition rate in both phases. Subsequent analysis of the evaluation revealed that the recognition rate is not influenced by the gender of the speakers. However, the gender of a rater is associated with the recognition rate. Sentence-wise perception rates have been presented in Tables 18 and 19. It can be seen that Neutral has achieved highest or second highest recognition rates for all sentences, and also all other emotions have achieved fairly good perception rate for all the sentences.

There are a few limitations of this study. Such as, only one mode of emotional expressions has been evaluated to simulate seven emotions. It will be interesting and desirable to carry out further research considering multiple modes of emotion expressions. For example, both video and audio expressions of emotion can be analyzed. During the evaluation of the corpus, only a subset of 280 files was given to each rater. Where in the ideal cases, each rater should rate all the audio files and each file should be rated several times by different users. In reality, rating all 7000 audio files by a rater in a single session is not possible. According to Ekman [48], compound emotions are formed by the combination of basic emotions (e.g. smugness is a combination of happiness and contempt). If an SER system is developed successfully for those basic emotions, in future it can be modified to recognize compound emotions correctly. It is important to select neutral sentences to confirm that the evaluation result is not biased by the semantic contents of the sentences. Purely neutral sentences are hard to express in target emotions. According to Burkhardt et. al. [14] nonsense sentences, fantasy words and speeches used in everyday life can meet this requirement. In that sense, Sentence 10 can be considered as fantasy words taken from fairy tales. Others can be considered as normal speeches taken from daily life. However, the expression of emotion of speech actually depends on context. It is also very important to ensure that these sentences can also be expressed in all of the target emotions.

## Conclusion

This paper presented the development and evaluation of a Bangla emotional speech corpus SUBESCO. It is an audio-only emotional speech database containing 7000 audio files which was evaluated by 50 validators. Several statistical methods were applied to analyze reliability of the corpus. Good perception rates were obtained for human perception tests (up to 80%). Reliability indices also showed quite satisfactory results. Two-way ANOVA was executed to analyse the effects of Gender and Emotion. The normality and homogeneity of data for these factors was also investigated using Jarque-Bera, Shapiro-Wilk, Levene, and Bartlette tests. A high rate of reliability and consistency of evaluation task shows that this corpus should be considered as a valuable resource for the research on emotion analysis and classification for Bangla language.

## Appendices

### Non-parametric tests

#### Emotion effects

ANOVA results showed that Emotion has a significant main effect on recognition rate. As the data does not show a normal distribution, a non-parametric test Kruskal-Wallis was conducted to investigate the distributions of emotion categories. The null hypothesis states that all emotions have the same population distribution. The Kruskal-Wallis results in Table 20 show that there was a statistically significant difference between the average emotion recognition rates for different types of emotion. The test statistics is H(6) = 167.09, p <.001 for Phase 1, and H(5) = 153.80, p <.001 for Phase 2.

**Table 20:** Kruskal-Wallis test statistics for Emotion.

Phase	df	H statistics	p-value	Reject H_*o*_
**Phase 1**	6	167.09	.000	Reject
**Phase 2**	5	153.80	.000	Reject

For post-hoc analysis of the Kruskal-Wallis test, non-parametric pairwise multiple comparisons were conducted using Dunn’s test at the significance level of.05. Tables 21 and 22 represent multiple pairwise Dunn’s test p-values for Phase1 and Phase 2, respectively. The test results are similar with that of pairwise t-test with a very few exceptions. It is clear that the emotion recognition rate is dependent upon the type of emotional state.

**Table 21:** Pairwise Dunn’s test for Phase 1 emotions.

	Anger	Disgust	Fear	Happiness	Neutral	Sadness	Surprise
**Anger**	1	.000	.000	.612	.000	.000	.000
**Disgust**		1	.020	.000	.000	.008	.001
**Fear**			1	.000	.000	.753	.247
**Happiness**				1	.002	.000	.000
**Neutral**					1	.000	.000
**Sadness**						1	.399
**Surprise**							1

**Table 22:** Pairwise Dunn’s test for Phase 2 emotions.

	Anger	Fear	Happiness	Neutral	Sadness	Surprise
**Anger**	1	.002	.001	.000	.000	.489
**Fear**		1	.000	.000	.000	.014
**Happiness**			1	.000	.048	.000
**Neutral**				1	.094	.000
**Sadness**					1	.000
**Surprise**						1

#### Rater’s gender effect

The Mann-Whitney U test was used to compare the population means of raters’ performances based on their genders. For Rater gender as depicted in Table 23, the test statistics were significant for both of the phases. Therefore, the null hypothesis was rejected indicating male raters’ data has a different distribution as compared to female raters’ data. A Chi-square test of independence was carried out to investigate whether there is an association between rater’s gender and the emotion recognition rate. A significant relationship was found between the two variables, which means the average recognition rate is not independent of the rater’s gender. For Phase 1, the Chi-square value is, *X*^2^(1, N = 14000) = 12.80, p <.001 and for phase 2, it is *X*^2^(1, N = 14000) = 11.13, p <.05. The overall Chi-square statistics for rater’s data are presented in Table 24. Likewise, from the results of the previous analyses of two-way ANOVA it was found that rater’s gender has a significant main effect on emotion recognition rate.

**Table 23:** Mann-Whitney U test for rater gender.

Subject	Emotion	Phase	N	df	Mann-Whitney U Statistics	p-value	Accept / Reject H_*o*_
Rater	All	Phase 1	14000	1	145.50	.001	Reject
		Phase 2	12000	1	186.000	.007	Reject

**Table 24:** Chi-square test for rater gender.

Subject	Emotion	Phase	N	df	Chi-square Statistics	p-value	Accept / Reject H_*o*_
Rater	All	Phase 1	14000	1	12.80	.000	Reject
		Phase 2	12000	1	11.13	.001	Reject

#### Speaker’s gender effect

The total numbers of correctly and incorrectly recognized emotions were calculated for each male and female speaker for both phases. The Mann-Whitney U test for speakers’ data was applied for both phases. The test statistics shown in Table 25 were not significant for all of the cases which indicates data relating to both genders have the same population distribution. The Chi-square test was also applied to find the association between speaker’s gender and overall emotion recognition rate. A Chi-square test yielded the Chi-square value of *X*^2^(1, N = 14000) = 2.22, p >.05 for Phase 1; and *X*^2^(1, N = 14000) = 0.06, p >.05 for Phase 2. Table 26 shows that Chi-square statistics are not statistically significant for any phase. That means, there is no evidence that a specific gender of speakers was dominant over another in terms of expression of more recognizable emotions. The outcome matches exactly with that of ANOVA analysis.

**Table 25:** Mann Whitney U test for speaker gender.

Subject	Emotion	Phase	N	df	Mann-Whitney U Statistics	p-value	Accept / Reject H_*o*_
Speaker	All	Phase 1	140	1	2418.000	.448	Accept
		Phase 2	120	1	1787.000	.474	Accept

**Table 26:** Chi-square statistics for speaker gender.

Subject	Emotion	Phase	N	df	Chi-square Statistics	p-value	Accept / Reject H_*o*_
Speaker	All	Phase 1	14000	1	2.22	.136	Accept
		Phase 2	12000	1	0.06	.803	Accept

## Supporting information

S1 Data Phase 1 evaluation measures for all 7000 stimuli of SUBESCO. It includes file name, speaker ID, speaker gender, rater ID, rater gender, intended emotion and rated emotion for each file.
(XLSX)

S2 Data Phase 2 evaluation measures for 6000 stimuli of SUBESCO. It includes file name, speaker ID, speaker gender, rater ID, rater gender, intended emotion and rated emotion for each file.
(XLSX)
